# Supplementary material for: A fault-intrusion-tolerant system and deadline-aware algorithm for scheduling scientific workflow in the cloud
Source: PeerJ Comput Sci. 2021 Nov 2;7:e747. doi: 10.7717/peerj-cs.747 (PMC8576554; doi:10.7717/peerj-cs.747)
Supplement: Supplemental Information 6 [file peerj-cs-07-747-s006.zip › Myproject1.1/docs/org/workflowsim/ClusterStorage.html]

ClusterStorage


JavaScript is disabled on your browser.


- Overview
- Package
- Class
- Use
- Tree
- Deprecated
- Index
- Help

- Prev Class
- Next Class

- Frames
- No Frames

- All Classes

- Summary:
- Nested |
- Field |
- Constr |
- Method

- Detail:
- Field |
- Constr |
- Method


org.workflowsim

## Class ClusterStorage

- java.lang.Object
- - org.cloudbus.cloudsim.HarddriveStorage
  - - org.workflowsim.ClusterStorage

- All Implemented Interfaces:
  :   Storage

  ---

    

  ```
  public class ClusterStorage
  extends HarddriveStorage
  ```

  ClusterStorage is an extention of HarddriveStorage and it is used as a local
  storage system of a vm

  Since:
  :   WorkflowSim Toolkit 1.0

- - ### Constructor Summary

    Constructors

    Constructor and Description

    `ClusterStorage(java.lang.String name,
    double capacity)`

    Initialize a ClusterStorage
  - ### Method Summary

    Methods

    | Modifier and Type | Method and Description |
    | --- | --- |
    | `double` | `getMaxTransferRate(java.lang.String destination)` Gets the bandwidth from this storage to the destination storage |
    | `void` | `setBandwidth(java.lang.String name, double bandwidth)` Sets the bandwidth between this storage to the destination storage |

    - ### Methods inherited from class org.cloudbus.cloudsim.HarddriveStorage

      `addFile, addFile, addReservedFile, contains, contains, deleteFile, deleteFile, deleteFile, getAvailableSpace, getAvgSeekTime, getCapacity, getCurrentSize, getFile, getFileNameList, getLatency, getMaxTransferRate, getName, getNumStoredFile, hasPotentialAvailableSpace, isFull, renameFile, reserveSpace, setAvgSeekTime, setAvgSeekTime, setLatency, setMaxTransferRate`
    - ### Methods inherited from class java.lang.Object

      `clone, equals, finalize, getClass, hashCode, notify, notifyAll, toString, wait, wait, wait`

- - ### Constructor Detail


    - #### ClusterStorage

      ```
      public ClusterStorage(java.lang.String name,
                    double capacity)
                     throws ParameterException
      ```

      Initialize a ClusterStorage

      Parameters:
      :   `name,` - name of this storage
      :   `capacity,` - capacity

      Throws:
      :   `ParameterException`
  - ### Method Detail


    - #### setBandwidth

      ```
      public final void setBandwidth(java.lang.String name,
                      double bandwidth)
      ```

      Sets the bandwidth between this storage to the destination storage

      Parameters:
      :   `name` - the destination storage
      :   `bandwidth` -


    - #### getMaxTransferRate

      ```
      public double getMaxTransferRate(java.lang.String destination)
      ```

      Gets the bandwidth from this storage to the destination storage

      Parameters:
      :   `destination` -

      Returns:
      :   bandwidth


- Overview
- Package
- Class
- Use
- Tree
- Deprecated
- Index
- Help

- Prev Class
- Next Class

- Frames
- No Frames

- All Classes

- Summary:
- Nested |
- Field |
- Constr |
- Method

- Detail:
- Field |
- Constr |
- Method
